# Oligodendrocyte Dysfunction in Amyotrophic Lateral Sclerosis: Mechanisms and Therapeutic Perspectives

**DOI:** 10.3390/cells10030565

**Published:** 2021-03-05

**Authors:** Stefano Raffaele, Marta Boccazzi, Marta Fumagalli

**Affiliations:** Department of Pharmacological and Biomolecular Sciences, Università degli Studi di Milano, 20133 Milan, Italy; stefano.raffaele@unimi.it (S.R.); marta.boccazzi@unimi.it (M.B.)

**Keywords:** oligodendrocytes, amyotrophic lateral sclerosis (ALS), remyelination, neurodegenerative disease, regenerative medicine, oligodendrocyte precursor cells (OPCs), GPR17 receptor, glial cells, monocarboxylate transporter 1 (MCT1)

## Abstract

Myelin is the lipid-rich structure formed by oligodendrocytes (OLs) that wraps the axons in multilayered sheaths, assuring protection, efficient saltatory signal conduction and metabolic support to neurons. In the last few years, the impact of OL dysfunction and myelin damage has progressively received more attention and is now considered to be a major contributing factor to neurodegeneration in several neurological diseases, including amyotrophic lateral sclerosis (ALS). Upon OL injury, oligodendrocyte precursor cells (OPCs) of adult nervous tissue sustain the generation of new OLs for myelin reconstitution, but this spontaneous regeneration process fails to successfully counteract myelin damage. Of note, the functions of OPCs exceed the formation and repair of myelin, and also involve the trophic support to axons and the capability to exert an immunomodulatory role, which are particularly relevant in the context of neurodegeneration. In this review, we deeply analyze the impact of dysfunctional OLs in ALS pathogenesis. The possible mechanisms underlying OL degeneration, defective OPC maturation, and impairment in energy supply to motor neurons (MNs) have also been examined to provide insights on future therapeutic interventions. On this basis, we discuss the potential therapeutic utility in ALS of several molecules, based on their remyelinating potential or capability to enhance energy metabolism.

## 1. Introduction

Oligodendrocytes (OLs) are highly specialized cells of the central nervous system (CNS) whose primary function is to form myelin, the lipid-rich structure organized in multi-layered sheaths around neuronal axons. Myelin sheath is important to assure long-term axonal integrity by means of metabolic and trophic support and is essential for rapid electrical nerve conduction [1]. 

In recent years, the impact of OL loss and myelin dysfunction in a variety of neurodegenerative disorders, including amyotrophic lateral sclerosis (ALS), has become increasingly clear. OL injury and demyelination, namely the damage to myelin structure, are now considered major contributors to the disability progression of neurological diseases [2,3]. These harmful events trigger a spontaneous repair process, known as remyelination, during which the oligodendrocyte precursor cells (OPCs), that persist in a quiescent state within the adult CNS [4,5], are activated, migrate, and differentiate to replace the OLs lost during pathological conditions [6]. However, the efficacy of this endogenous repair process is generally low, leading to permanent deficits and functional impairments [7]. 

Here, we summarize the crucial functions of adult OPCs in the CNS, starting from their ability to myelinate axons and to regenerate disrupted myelin after injury, necessary to preserve efficient impulse conduction and trophic support to neurons. The reactivity of these cells is also discussed based on recent data suggesting that their responsiveness to myelin damage is markedly dependent on their heterogeneity [8]. We also report recent evidence showing the capability of OPCs to sense and react to inflammation, playing a critical role in the modulation of the immune-inflammatory response which accompanies many neurological diseases [9]. In particular, we discuss the role of oligodendroglia in ALS, based on a growing number of studies revealing that OL injury is a crucial contributing factor ultimately leading to neuronal degeneration and pathology progression. Finally, we highlight currently evaluated drugs for OL and myelin repair therapies that might potentially serve as therapeutic approaches in ALS.

## 2. Oligodendrocyte Functions in Adult CNS

### 2.1. Myelin Generation and Remyelination

**Figure 1:**
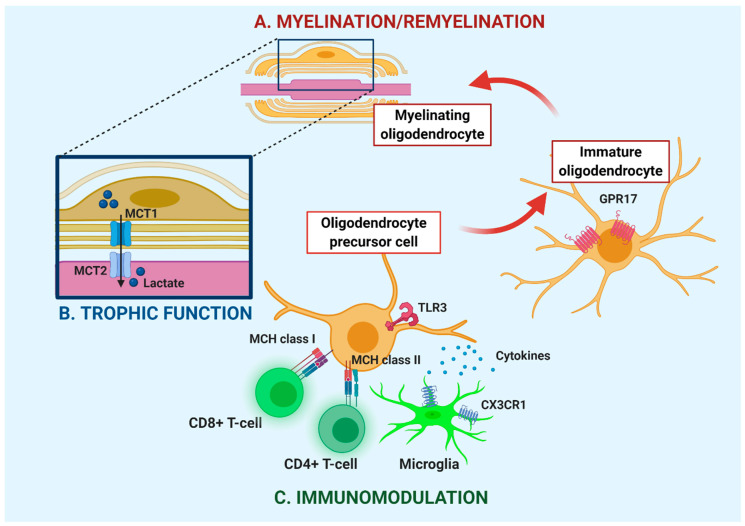
Functions of oligodendrocyte (OL) lineage cells in the adult central nervous system (CNS). Oligodendrocyte precursor cells (OPCs) become mature and sustain myelination and remyelination during development or in response to injury (**A**). Mature myelinating OLs release lactate produced by glycolysis into the periaxonal space through monocarboxylate transporter (MCT) 1. Neurons can in turn take up lactate through the MCT2 to support mitochondrial ATP synthesis (**B**). Finally, OPCs in the disease state are also known to express major histocompatibility complex (MHC) class I and II and several cytokines which enable them to modulate T-cells and microglia, thus affecting the immune response (**C**).

Classically, the primary and most investigated role attributed to OLs is to form myelin, a multilamellar fatty membrane, mainly composed by glycosphingolipids and cholesterol, that enwraps axons ensuring their insulation and the saltatory conduction of nerve impulses [10] (Figure 1A). The formation of myelin is a complex process during which OPCs become mature OLs through a highly regulated program of differentiation [11]. OPCs are generated from radial glia cells in multiple localized areas during embryogenesis [12,13,14]. In the developing murine spinal cord, OPCs initially arise around embryonic day 12.5 (E12.5) in the ventral ventricular zone (VZ) and migrate all through the spinal cord before differentiating into myelin-forming OLs [15,16]. Later, around E15.5, some OPCs start to be generated in the dorsal VZ, contributing 10–15% of the final OL population [12,13,14]. Regarding mouse forebrain, three sequential waves of OPCs are generated from different parts of the telencephalic VZ with a temporal ventral-to-dorsal progression [17]. 

In rodents, OPCs differentiate during the first two postnatal weeks, whereas in humans they mostly develop between 23 and 37 weeks of gestation [18] and CNS myelination gradually increases in a defined topographic and temporal sequence within the first two decades of life [19]. Importantly, OPCs are still present throughout the adult grey (GM) and white matter (WM) parenchyma where they constitute approximately 6% of the CNS total cell number [5,20]. These cells are engaged into maturation to sustain myelin remodeling [21], and in response to motor learning [22,23]. Furthermore, adult OPCs represent an important source of remyelinating cells able to generate new myelinating OLs during the repair of demyelinated lesions (Figure 1A), together with surviving mature OLs which have been recently shown to be able to regenerate new myelin sheaths [24]. The OPC maturation process comprises a complex sequence of events that can be simplified as follows: migration and proliferation of OPCs, differentiation of OPCs into myelinating OLs, initiation of membrane outgrowth and axonal wrapping, and rapid expansion of the myelin sheath and node formation [25,26]. A large number of existing studies in the broader literature have examined the role of several intrinsic (cell autonomous) and extrinsic (also defined as environmental or non-cell autonomous) factors that finely regulate the initial steps of myelin generation (migration, proliferation, and differentiation of OPCs; extensively reviewed in [11,27]) and that are also involved in remyelination [3,28]. 

An important regulator of OPC maturation, both during early development and at adult stages, is the G protein-coupled receptor (GPCR) GPR17 (Figure 1). This receptor responds to both extracellular nucleotides, mainly uridine-5’-diphosphate (UDP) and UDP-glucose, and cysteinyl-leukotrienes [29], endogenous signaling molecules involved in both inflammatory response and in the repair of CNS lesions. It is highly expressed during the transition from OPCs to immature OLs [30,31,32], but, after this stage, it has to be downregulated to allow cells to complete their maturation toward myelinating OLs [32,33]. In detail, the receptor is present in two pools of progenitors labeling two distinct stages of OL differentiation: the early stage of proliferative OPCs expressing markers like neural/glial antigen 2 (NG2), ganglioside epitope recognized by the A2B5 antibody (A2B5), and platelet-derived growth factor receptor-alpha (PDGFRα), and the subsequent phase of more ramified, immature OLs, characterized by NG2 downregulation, and by the expression of more advanced markers like the sulfatides O4 and O1. Accordingly, GPR17-expressing OPCs are already present in mice at birth, their number increases over time, reaching the highest expression level at postnatal day 10 (P10), before the peak of myelination, and then declines in the adult brain [34]. Interestingly, receptor obliteration with small interfering RNAs in early OPCs profoundly reduces their ability to generate mature OLs, suggesting a pivotal role of GPR17 in the initial phases of the differentiation process [32]. On the other hand, loss of GPR17 at advanced differentiation stages is necessary to enable cells to complete terminal maturation. Consistently, GPR17 forced overexpression in differentiating OPCs keeps cells at an immature phenotype that does not express 2′,3′-Cyclic-nucleotide 3’-phosphodiesterase (CNPase), a relatively advanced OL marker. In line with this finding, myelinogenesis was found to be defective in transgenic mice overexpressing GPR17 under the promoter of CNPase [33]. It has also been observed that, under conditions where terminal OPC maturation is impaired (such as demyelinating diseases or following treatment with the mTOR inhibitor rapamycin), GPR17 is markedly upregulated [35,36,37]. Finally, single-cell RNA-sequencing (RNAseq) analysis aimed at unveiling the existence of a wide heterogeneity in oligodendroglial subpopulations within the brain, reported GPR17 expression only in three clusters (out of 12 distinct cell subpopulations belonging to the OL lineage) defined as “differentiation committed precursors” [38]. These recent data further corroborated the central role of GPR17 in orchestrating oligodendroglial differentiation and myelination. 

However, what governs OL decision to form initial sheaths and which axons to myelinate or remyelinate is not yet entirely clear. A lot of evidence supports the hypothesis that axonal parameters such as caliber, presence or absence of inhibitory signals, and activity-dependent secreted factors can regulate ensheathment fate [6,39,40,41], suggesting that myelination is the result of a highly orchestrated interaction between neurons and OLs. In this respect, Bechler and colleagues showed that myelinating cells of the CNS are able to sense axon diameter and in turn increase their sheath length on larger fibers. Of note, OLs from cortex and spinal cord form sheaths of different lengths, with spinal cord OLs generating longer sheaths than cortical OLs [42]. In addition, a very elegant and recent work carried out in zebrafish spinal cord identified two distinct OPC populations, distinguishable by the diverse localization of their soma and with different abilities to myelinate. Time-lapse imaging studies showed that the vast majority of OPCs within axo-dendritic areas begin to differentiate and to wrap axons within 24 hours of investigation, whereas none of the OPCs with their soma in the neuron-rich areas does so during the same time frame. Interestingly, the GPR17 receptor was found to be strictly present in cells localized in an axon-enriched environment and potentially able to myelinate axons, while it is virtually absent in OPCs housed in proximity of neuronal somas, which never differentiate but seem to be more involved in network formation and synaptic activity [43]. Of note, axonal electrical activity has also been demonstrated to be crucial for remyelination in different experimental models characterized by myelin damage [44,45].

#### The Heterogeneity of OLs in Response to Demyelination

Heterogeneity is a well-known characteristic of the majority of the cell types in the body, including those of the CNS. First described in neurons, it is now widely accepted that also glial cells are characterized by a great diversity. In particular, in vivo clonal analysis and single-cell RNAseq have revealed both molecular and behavioral heterogeneity among OL lineage [8,46,47,48].

To describe diversity among OLs, it is necessary to consider their developmental heterogeneity, which is primarily linked to their ventral or dorsal origin as described above. However, the use of genetic ablation strategies, to individually eliminate each OPC population arising in the forebrain during development, suggested that different embryonic origins do not correspond to functional heterogeneity [17]. Furthermore, in both spinal cord and corpus callosum, OPCs and their progeny have indistinguishable electrical properties irrespectively of their origin. During adulthood, OPCs display a certain level of diversity which mainly depends on their anatomical/regional location in the GM or WM. Indeed, fate mapping studies revealed that OPCs proliferate slower in the GM compared with the WM and produce fewer mature cells [49]. This seems to be more linked to intrinsic differences rather than depending on the environmental niche [50]. Also in humans, the kinetics of OL generation and turnover differ between these two structures, with GM OLs having a longer expansion phase and a higher turnover compared to WM OLs [19]. Furthermore, GM and WM OPCs differ in terms of electrophysiological capacities (i.e., membrane properties and K^+^- and Na^+^-channel expression profile) [51].

Interestingly, it has been suggested that the developmental origin of OPCs influences their regenerative properties in adulthood following a demyelinating injury. Indeed, after lysolecithin-induced focal demyelination in the ventral funiculus of the spinal cord or in the corpus callosum, dorsally derived OL lineage cells dominate the remyelination response in both regions. However, the dorsally derived cells showed an accelerated age-related impairment in their ability to differentiate into myelinating OLs compared to their ventrally derived counterparts [46]. Moreover, a recent paper shows that reduced sonic hedgehog (SHH) signaling from VZ progenitors results in a severe diminishment of ventrally derived OPCs but normal numbers of motor neurons (MNs) in the postnatal spinal cord. As a consequence, dorsal OPCs populate the entire spinal cord leading to an increased OPC density in the ventral horns. However, these OPCs display morphological and functional abnormalities similar to those found in dysfunctional cells surrounding degenerating MNs in a mouse model of ALS [47]. These results suggest that spinal cord dorsal OPCs are not equivalent to ventral OPCs in their ability to interact with surrounding MNs upon injury [47].

The advent of single-cell RNAseq allowed the characterization of the OL cell lineage at the transcriptome level with unprecedented precision and revealed an unexpected heterogeneity among them [52]. As already explained above, during embryogenesis, OPCs populate the spinal cord in a specific pattern, in which ventral OPCs appear early and spread uniformly throughout the cord, whereas dorsal OPCs arrive later and remain mainly in the dorsal and dorsolateral funiculi during postnatal age. However, later in life, ventrally derived cells are replaced by their dorsally derived counterparts, positioned so that the corticospinal and rubrospinal tracts become myelinated mainly by dorsally derived OLs [53]. In this respect, a pioneering single-cell RNAseq analysis showed a clear temporal segregation of cells at E13.5 and P7, whereas, after this developmental stage, OPCs from different CNS regions, such as the brain and spinal cord, present a comparable gene expression profile. Thus, even though they are derived from different precursor domains, in unchallenged animals, these cells converge towards similar transcriptional states. Initial stages of differentiation were comparable also across the juvenile CNS, since similar populations were also observed in all regions in juvenile mice between P21 and P60 [38]. On the contrary, different regions of the CNS were populated by diverse mature OLs, with some populations present throughout the analyzed area and others enriched in specific regions in the adult brain [38]. Although gene profiling analysis concluded that it was not possible to identify region- or age-specific subpopulations of OPCs, profound differences in the proliferative capacity between juvenile and adult OPCs were observed (with 16% of the juvenile OPCs and only 3% of the adult counterparts being in the cell cycle). This should not be surprising, since it is well established that adult OPCs cycle less intensely with age and differentiate with a progressively slower rate in postnatal and adult life. Thus, these aspects should also be taken into account when interpreting single-cell RNAseq data, especially in a disease context.

As mentioned above, a recent work identified two functionally distinct populations of OPCs in the zebrafish spinal cord. One subgroup has been found to establish elaborate networks of processes and to exhibit remarkably high calcium activity. These cells rarely differentiate into mature OLs, but retain the capacity to divide in an activity- and calcium-dependent manner, producing a second OPC subpopulation characterized by higher process motility and reduced calcium signaling, which readily differentiates into myelinating OLs [43].

The heterogeneity of OLs has also emerged in the context of a demyelinating disease. A clear segregation of cells between control and Experimental Autoimmune Encephalomyelitis (EAE) mice, the most commonly used model of multiple sclerosis (MS), has been demonstrated, with specific EAE-enriched OPCs and mature OLs populations that expressed unique genes which were completely absent or present at a very low level in healthy controls [8]. Interestingly, a very recent paper published by the same group demonstrated that, in a mild model of traumatic spinal cord injury (SCI), two transcriptionally diverse populations of mature OLs respond differently to injury [48]. Indeed, one population increased in corticospinal tract from juvenile to adulthood and pronominally contributed to the OL lineage at the injury site after SCI, whereas the other one was preferentially identified in sensory tracts of the spinal cord and participated in the OL lineage in regions of Wallerian degeneration specifically during the chronic phase [48]. This seems to suggest that different OPC populations might have distinct roles during regenerative phases after injury, having unique abilities to communicate with lesioned MNs, as already suggested for ventral and dorsal OPCs [47].

Single-nucleus RNAseq from WM areas of post-mortem human brain from patients with MS and from unaffected controls identified subclusters of OLs in control human WM, some with similarities to mouse, and defined new markers for these cell states. Notably, some subclusters were underrepresented in MS tissue, whereas others were more prevalent. These differences in mature OL subclusters may indicate different functional states of OLs in MS lesions. Interestingly, similar changes in normal-appearing WM have also been observed [54].

In conclusion, single-cell RNAseq analysis reveals a high heterogeneity among OLs that seems to be further exacerbated in a disease context. The selective involvement of specific OPC populations after injury, and the decreased proliferation and differentiation capability of these cells with age and disease progression, might lead to the “exhaustion” of the reactive pool of OPCs and to the selection of defective and immature cells. Although research in this direction is just in its infancy, it would be important to fully elucidate the functional contribution of each OL subclusters to myelin disorders, including ALS, and to develop novel targeted therapeutic approaches. 

### 2.2. Trophic and Metabolic Support to Neurons

Lessons from classical demyelinating diseases like MS suggest that axonal preservation and more benign disease progression are not only dependent on myelin integrity enabling fast nerve impulse transmission but also on a direct function of OLs in sustaining axonal integrity and neuronal survival [55]. Recent data indicate that distinct metabolic pathways (glycolysis, fatty acid synthesis, mitochondrial and peroxisomal fatty acid β oxidation) active in OPCs and in mature OLs may influence their capability to proliferate, differentiate and to provide trophic support to the associating neurons [56]. Due to their role in action potential transmission, neurons are highly energy demanding cells and about 2/3 of the total brain ATP production is spent on the membranous ion pump Na^+^/K^+^-ATPase transporting Na^+^ out and K^+^ into the cell [57]. On the other hand, they do not have a high amount of capacity to store glycogen and, in most long axons, the access to extracellular glucose is limited by the presence of myelin [58]. For these reasons, it should not be surprising if axons derive some metabolic energy directly from other cells. OLs predominantly utilize glycolysis for ATP production, and are considered to fulfill high energy demands of axons by giving them the lactate derived from glucose [59,60]. OLs could take up glucose using the glucose transporter 1 (GLUT1) or through gap junction composed of connexin (Cx). In particular, OPCs take up glucose from the extracellular microenvironment through Cx-hemichannels (i.e., Cx29 and Cx47) [61]; on the contrary, the source of glucose for mature OLs is from astrocytes through connexin-based gap junction channels (two oligodendroglial proteins, Cx32 and Cx47 form heteromeric channels with astrocyte Cx30 and Cx43, respectively). Evidence suggests that both connexin-hemichannel and connexin-based gap junction channels are important for oligodendroglial functions. The pharmacological inhibition of both Cx29 and Cx47-hemichannels activity in OPCs causes reduced glucose concentrations and decreases proliferation [61]. Of note, reduced expression of the oligodendroglial connexins Cx32 and Cx47 has been reported both in EAE and in MS patients [62,63,64]. In addition, mutations of Cx32 induce Charcot–Marie–Tooth disease type 1X (CMT1X), an inherited peripheral neuropathy, and Cx47 mutation is associated with a Pelizaeus–Merzbacher-like disease (PMLD), a severe childhood-onset leukodystrophy [65]. Interestingly, a recent study supports a mechanism in which oligodendroglial glutamate NMDA receptors (NMDARs) stimulation by neuronal activity leads to GLUT1 mobilization and incorporation into the myelin compartment. GLUT1 upregulation occurs within minutes and augments glucose import, stimulating lactate release [66]. 

As already stated before, mature OLs use part of their glycolysis product lactate to fuel the axonal compartment for mitochondrial ATP production. In particular, the monocarboxylate transporter 1 (MCT1), the most abundant lactate transporter in the CNS, is highly enriched in OLs and is responsible for the release of lactate produced by glycolysis into the periaxonal space (i.e., a 10 to 20 nm wide space between the axon and its surrounding myelin sheath). Neurons can in turn take up lactate through the MCT2 to support mitochondrial ATP synthesis [59,60] (Figure 1B). OL-specific MCT1 inhibition with antisense oligonucleotides or a specific pharmacological inhibitor (MCT1i) in organotypic spinal cord slice cultures causes axonal damage and neuron loss. Of note, loss or blockade of MCT1 was not toxic to OLs themselves and neuronal death was rescued by adding L-lactate to the culture medium. Furthermore, in vivo experiments aimed at silencing MCT1 expression either globally or selectively within OLs resulted in severe axonal injury and MN death [60]. Of note, a recent study reported that MCT1 protein levels in OLs are gradually reduced during physiological aging. In conditional knockout animals, OL-specific genetic ablation of MCT1 did not affect axonal integrity during early development, while it produced axonopathy and hypomyelination in late adulthood. These results indicate that lactate provision to MNs by OLs becomes progressively important with aging, when MCT1 deficiency may increase the susceptibility to neurodegenerative diseases [67]. Accordingly, in ALS rodent models and patients, the expression levels of this transporter in mature OLs are significantly reduced, suggesting that alterations in oligodendroglial MCT1 may contribute to MN degeneration ([60]; see also below). 

Another paper published in the same year by a different group has provided evidence that CNS neurons can survive without degeneration thanks to the glycolytic metabolites transferred from OLs to axons [59]. They used conditional Cox10 (protoheme IX farnesyltransferase) knockout mice, in which OLs and Schwann cells fail to assemble stable mitochondrial cytochrome c oxidase leading to ineffective mitochondrial respiration. Mutant mice exhibit severe neuropathy with dysmyelination, muscle atrophy, and paralysis in the peripheral nervous system. In contrast, in the adult CNS, no signs of demyelination, axonal degeneration, or secondary inflammation were observed. However, brain lactate measured by magnetic resonance spectroscopy was increased in both cortex and WM in mice under isoflurane anesthesia (i.e., reduced metabolic activity in neurons) likely as a result from increased exports of lactate from OLs forced to utilize non-oxidative metabolism due to mitochondrial mutations. Interestingly, the levels of lactate rapidly returned to that of controls after discontinuing anesthesia, possibly due to the restored neuronal activity and the rapid uptake by axons [59].

### 2.3. Immunomodulatory Properties

Neuroinflammation is a common pathological feature of many neurodegenerative diseases, including ALS. Both resident (mainly astrocytes and microglia) and circulating immune cells act as sentinels of the CNS in order to maintain tissue homeostasis. During chronic disease, these cells finely orchestrate the inflammatory response participating not only in the cytotoxic damage, but also in immune regulation and injury resolution. In this scenario, it is now emerging that OLs are not simply passive witnesses or inert victims in neuroinflammatory processes, but they may play themselves an important active role in tissue damage and repair by assuming an immunological profile. 

Numerous reports show that OLs can express a wide range of cytokines and chemokines (including IL1-β, CXCL10, CCL2, CCL5 and CXCL9, reviewed in [68]), and also several receptors for immune related molecules, which enable them to sense inflammation and to react [69]. 

Recently, in an in vivo mouse model which mimics inflammation-mediated WM injury of preterm born infants by intraperitoneal injection of IL-1β, O4^+^ immature OLs and PDGFRα^+^ OPCs showed a different sensitivity to neuroinflammation. This was demonstrated by the greater upregulation of *Tlr3*, *IL-1β*, *IFN-β*, *Ccl2* and *Cxcl10* in immature OLs as compared to OPCs in the IL-1β treated animals. Results also showed that the inflammatory response of OLs can play an autonomous role in blocking their own differentiation and in shaping the response of microglia [70]. 

The cross-talk between OLs and microglia was further confirmed by a couple of recently published papers, describing that OPCs are essential for the maintenance of microglia in a quiescent state in several animal and disease models [71,72]. Indeed, OPC depletion using two potent inhibitors of PDGF in cultured brain slices have been shown to downregulate the microglia homeostatic signature [71]. This was observed also in vivo in several transgenic mice with ablated OPCs (PDGFRα-CreER/iDTR, NG2-CreER, PDGFRαflox/flox and DTRNG2) [71,72]. It is noteworthy that, in addition to ensuring microglial homeostasis, OPCs also controlled the intensity of microglial activation after lipopolysaccharide (LPS) administration in vivo. Gain and loss-of-function studies further demonstrated that OPC regulation of microglial homeostasis is mediated by the release of transforming growth factor-beta 2 (TGF-β2) by OPCs, which in turn regulates the expression of CX3CR1, a microglial receptor that binds to fractalkine promoting a neuroprotective response [72] (Figure 1C). Interestingly, deficiency of OPCs has been shown to contribute to neuroinflammation and neurodegeneration in the 1-methyl-4-phenyl-1,2,3,6-tetrahydropyridine (MPTP)-induced mouse model of Parkinson’s disease (PD). Indeed, the loss of tyrosine hydroxylase–positive dopaminergic neurons and the increase in the number of microglia in the substantia nigra were significantly worse in OPC depleted mice following MPTP exposure compared to MPTP-treated control animals [72].

Recent work indicated that, after neural injury and retinoic acid receptor-β activation, OPCs, migrating into the lesion site, synthesize retinoic acid, which is then encapsulated into extracellular vesicles and delivered to neurons, providing a permissive cue for neurite growth and regeneration [73]. In addition, following nerve injury, it has been observed that OPC interaction with damaged MNs is needed for efficient elimination of glutamatergic (vGlut1) synapses [47]. Thus, given the very well-known role of microglial cells in synaptic pruning, it can be speculated that OPCs might also indirectly participate in this process by modulating microglia functional states and by orchestrating their interaction with target neurons. 

Of note, recent single-cell RNAseq of OPCs during EAE [8] and in MS tissue [54] confirms what already observed in the early ’90 [74,75,76]: OL lineage cells express genes involved in immunoprotection and in antigen processing and presentation via major histocompatibility complex class I and II (MHC-I and -II). In particular, direct contact with immune cells such as T-cells, and exposure to cytokines as IFN-γ, allow OPCs to express MHC I and II and present antigens to CD8^+^ and CD4^+^ T cells, respectively [8,77,78] (Figure 1C), leading to their own activation (increased survival, proliferation, and cytokine production).

## 3. Oligodendrocyte Dysfunction in ALS

ALS is a neurodegenerative disorder characterized by the progressive loss of MNs in the cerebral cortex and spinal cord, leading to muscle atrophy and death by respiratory paralysis within 3–5 years after the first symptoms [79]. The majority of ALS cases are sporadic (ALS occurs in people without a family history of the disease) [80], while approximately 10% of ALS-affected individuals have at least one other family member with ALS. Both familial and sporadic cases are associated with pathogenic variants in known ALS-linked genes, including superoxide dismutase 1 (SOD1), TAR DNA binding protein (TARDP) encoding for TDP-43, hexanucleotide expansion of chromosome 9 open reading frame 72 (C9orf72), fused in sarcoma (FUS), and optineurin (OPTN) [79,81]. Familial and sporadic ALS share common pathogenetic mechanisms, including (i) aggregation of misfolded or mutated proteins, most commonly SOD1, TDP-43 and FUS, causing neurodegeneration directly or through activation of detrimental neuroinflammatory response; (ii) alteration of nuclear RNA transport, resulting in neurotoxicity due to perturbation of gene splicing and sequestration of RNA-binding proteins within the nucleus; (iii) impairment of cytoskeletal dynamics affecting axonal stability, transport and growth [79]. In addition, pathological alterations of neuronal excitability and synaptic plasticity have been described in the different types of ALS, possibly increasing the vulnerability of MNs to excitotoxicity [82].

Despite disease clinical manifestation of ALS being clearly associated with MN degeneration, it is now well established that its pathogenesis is also dependent on non-cell autonomous mechanisms, meaning that other cell types contribute to disease progression by triggering or aggravating damage to MNs (reviewed in [83]). In particular, recent reviews have summarized the neuroinflammatory role of astrocytes and microglia in ALS, unveiling their critical involvement in MN degeneration and boosting new interest on the potential benefits of anti-inflammatory and immunomodulatory agents in ALS clinical management [84,85].

In parallel, pathological alterations of the WM have been detected in ALS patients and animal models, suggesting that OLs and myelin structures are also affected during disease progression. Accordingly, a recent genome-wide study demonstrated that OLs incorporate a significant proportion of ALS genetic risk, supporting the hypothesis that OL dysfunction may be a primary contributing factor to disease pathogenesis [86]. In the following paragraphs, we will describe the role of OL lineage cells in the pathogenesis of ALS. 

### 3.1. Dysmphorphic OLs during Disease Progression

Starting from the first data reporting the presence of pathological protein aggregates of mutant SOD1 in periaxonal OL extensions in the SOD1 G93A murine model of ALS [87], signs of myelin damage have been described at a late disease stage in spinal cord ventral horn GM [88] and WM [89], as well as in upper structures of the brain [90,91]. Notably, post-mortem histological analyses detected degenerative changes in OLs, namely TDP-43 inclusions, in the spinal cord GM of sporadic ALS patients [92]. OL pathology was well reproduced in mutant SOD1-bearing mice, in which the number of degenerating OLs was found to dramatically increase with disease progression. These cells were characterized by abnormal morphology with thickened cell body and cleaved caspase 3 immunoreactivity (a marker of apoptosis), and were surrounded by clusters of activated microglia phagocytosing cell debris [88,92]. Abnormalities of myelin structures have been reported in the spinal cord of SOD1 G93A mice, as well as in motor cortex and spinal cord GM of ALS patients [88]. Accordingly, reduced levels of myelin basic protein (MBP) were found in the spinal cord of SOD1 G93A mice [92]. Interestingly, the extent of WM degeneration, measured using diffusion tensor imaging (DTI) techniques, was recently demonstrated to correlate with disease severity and progression in patients [93]. A progressive reduction of fractional anisotropy (FA), a measure of WM fiber density, axonal diameter, and myelination, was found in the frontal lobes and corticospinal tract of ALS subjects compared to healthy individuals. Reduced FA correlated with patient’s clinical disability, assessed using the ALS Functional Rating Scale-Revised (ALSFRS-R), suggesting that damage to myelin structures critically contributes to the development of functional deficits [93]. Intriguingly, degeneration of mature OLs and myelin defects were found to start at pre-symptomatic phase in SOD1 G93A mice spinal cord [88,89,94,95,96], suggesting that myelin disruption anticipates MN degeneration and directly contributes to disease exacerbation. In line with this, we recently observed the presence of OL abnormalities in the lumbar spinal cord of SOD1 G93A mice at very early developmental stages (P7-10), including increased levels of immature markers like NG2 and GPR17 and reduced density of CC1^+^ mature cells [96]. However, despite the growing understanding of oligodendrocyte maturation in the developmental myelination, the underlying molecular mechanisms or factors involved in the context of ALS disease remain poorly investigated.

### 3.2. Defective Response of OPCs to Degeneration of Mature OLs in ALS

**Figure 2:**
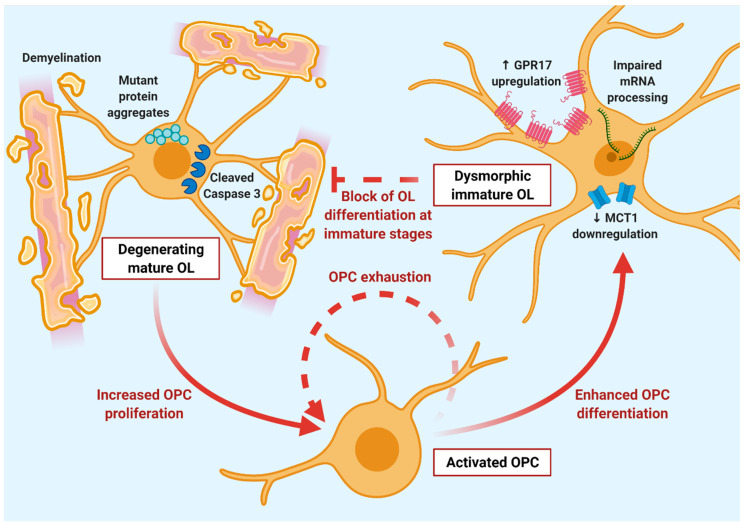
Oligodendrocyte (OL) dysfunction in ALS. In experimental models and patients affected by ALS, pathological alterations of myelinating OLs have been observed, i.e., the formation of toxic aggregates of ALS-related mutant proteins. Consequently, OLs start to degenerate, as confirmed by typical signs of apoptosis like cleaved caspase 3 reactivity, leading to axonal demyelination. In response to myelin disruption, oligodendrocyte precursor cells (OPCs) increase their proliferation rate and undergo differentiation to replace dying cells with new mature OLs. However, these newly-formed OLs are characterized by abnormal upregulation of the GPR17 receptor, likely contributing to inhibit their terminal maturation, and by the downregulation of the monocarboxylate transporter MCT1, required for supplying lactate and other nutrients to motor neurons (MNs). A defective processing and trafficking of myelin-related mRNAs was shown to further contribute to impaired remyelination. Hence, these alterations result in the generation of dysmorphic and immature OLs, unable to remyelinate axons and to restore their trophic support to MNs, and in the exhaustion of the reserve pool of OPCs due to increased turnover rate.

In response to myelin damage, OPCs increase their proliferation rate and migratory ability and start to differentiate in an attempt to replace degenerating myelin-forming OLs. Indeed, even after the extensive loss of myelinating cells, no changes in the total number of CC1^+^ OLs were found in the spinal cord of SOD1 G93A mice with respect to control littermates, suggesting an increased turnover in response to the degeneration of mature cells [88,92]. This was confirmed by fate mapping experiments using reporter mouse lines for early OPCs and mature OLs crossed with SOD1 G93A background, demonstrating increased OPC proliferation and differentiation during disease progression in the spinal cord of SOD1 G93A mice [90,94,97]. However, further experiments showed that, in SOD1 G93A mice, OPC regenerative response was defective, contributing to MN loss. Indeed, the capability of OPCs to differentiate into fully mature and functional cells was found to be impaired in ventral GM of ALS mice, resulting in the presence of immature and dystrophic newly-formed OLs, unable to provide an efficient remyelination and metabolic support to neurons [88,92]. In this respect, the protein levels of the oligodendroglial lactate transporter MCT1, required to shuttle nutrients from OLs to MNs, were reported to progressively decrease in the spinal cord of SOD1 G93A mice until the end stage of the disease [60,92]. Of note, a reduction of MCT1 protein levels, quantified by Western blotting, has been reported also in the motor and frontal cortex of sporadic ALS patients [60]. This effect was demonstrated to be dependent, at least in experimental models, on the inhibitory effect of mutant SOD1, which blocks MCT1 expression at the post-transcriptional level, since MCT1 mRNA expression was not altered in SOD1 G93A mice compared to controls [92]. Importantly, oligodendroglial MCT1 deficiency has been shown to contribute to the loss of MNs, as MCT1 knockout mice developed widespread axonopathy, and specific silencing of MCT1 in OLs, using genetic or lentiviral approaches, induced axonal injury [60,67]. However, the enhancement of MCT1 expression in spinal cord WM OLs of mutant SOD1 mice, achieved using viral gene delivery, did not result in improved MN viability, survival rate or functional outcome [98]. These results highlight a significant impairment of OL functions in ALS (Figure 2), which affects not only their capacity to myelinate axons, but also their essential contribution to energy metabolism of MNs. Based on the recent findings showing a wide heterogeneity of OPC subpopulations, each one probably addressing specific functions (see Section 2.1.1), it could be postulated that some cell clusters may be present at pre-symptomatic stages with the proper genetic background to efficiently replace dysfunctional OLs. However, as damage progresses, regeneration-committed OPCs are probably overwhelmed by the elevated turnover, leading to the exhaustion of this reserve pool and to the accumulation of dysfunctional OLs described at the end-stage of the disease. Thus, a detailed characterization of the abnormalities underlying defective OPC differentiation in ALS, together with appropriate longitudinal studies evaluating OPC reactivity at single-cell resolution, will help to identify novel targets for preventing MN degeneration.

### 3.3. Possible Mechanisms Contributing to OL Pathology and Dysfunction in ALS

#### 3.3.1. ALS-Causing Mutant Genes

The presence of mutant ALS-associated genes, such as SOD1, was found to be harmful to OLs, and indeed early genetic ablation of mutant SOD1 from oligodendroglial cells significantly delayed disease onset and prolonged overall survival [88]. On the contrary, cell-specific overexpression of mutant SOD1 in mature OLs, in a zebrafish model, directly induced demyelination, promoting MN degeneration and reproducing a pathological phenotype similar to mouse models expressing mutant SOD1 in all cell types [99]. Notably, MN-specific expression of mutant SOD1 was demonstrated to not cause neurodegeneration, for which the contribution of surrounding non-neuronal cells is required [97,100]. SOD1-dependent OL loss has been shown to induce MN death via two distinct mechanisms. The first one is linked to defective lactate release due to MCT1 downregulation, whereas the second one is lactate-independent and involves cell–cell contact between axons and enwrapping OLs [101]. Interestingly, knockdown of mutant SOD1 in mature OLs using short hairpin RNAs was not able to reduce MN toxicity, while it effectively restored lactate production and MN survival when performed in immature precursors [101]. These data suggest that the MN-toxic phenotype of ALS mature OLs progressively develops during OPC differentiation in the presence of mutant SOD1 and becomes fatally irreversible in fully differentiated cells [101].

Similarly, other mutant proteins involved in ALS pathogenesis, including TDP-43 and FUS, have been demonstrated to trigger OL degeneration through a specific modification of their proper function, or more unspecifically by their increased tendency to form toxic aggregates [91,102,103]. Notably, the presence of protein inclusions has been detected not only in familial ALS patients, but also in subjects affected by sporadic forms of the disease [104,105]. 

In addition, loss-of-function mutations of the polyubiquitin-binding protein OPTN have been recently associated with both familial and sporadic forms of ALS [106,107]. Due to its capacity of interacting with different binding partners, OPTN has been involved in several biological processes, including neuroinflammation, necroptosis, and autophagy [108]. All of these pathways are known to be dysregulated in ALS patients; however, the exact mechanism by which OPTN mutations contribute to disease progression has not been fully defined yet. In this respect, genetic ablation of OPTN in the whole body or specifically in OLs or microglia, but not in MNs and astrocytes, has been recently shown to induce progressive axonal pathology and myelin abnormalities in the mouse spinal cord, and consequent hindlimb disability, resembling those found in ALS patients at early stages of the pathology [109]. At the basis of these detrimental effects, OPTN deficiency has been demonstrated to increase the vulnerability of OLs to necroptosis, a form of caspase-independent cell death involving the activation of receptor-interacting kinase 1 (RIPK-1) [109]. It is worth noting that similar findings were later confirmed also in the spinal cord of SOD1 G93A mice and ALS patients, suggesting that OL necroptosis may contribute to axonal pathology, independently of OPTN mutations [109].

From these data, ALS-related genetic mutations appear to be intimately linked to OL dysfunction, even in sporadic forms of the disease. However, since ALS is a complex and multifactorial disease, it is likely that other mechanisms also contribute to the generation of dysfunctional OLs.

#### 3.3.2. Impaired mRNA Processing

A recently emerging aspect of OL dysfunction in ALS involves alterations of mRNA metabolism [110,111]. Indeed, effective myelination by OLs strongly depends on local synthesis of many proteins, including MBP, which requires the long-distance delivery of mRNAs from the nucleus to cell processes [110]. Most of the genes mutated in ALS are involved in RNA trafficking, including TDP-43, FUS, and C9orf72 [110]. On this basis, myelin defects occurring in ALS may depend on impaired transport of mRNAs and translation into functional myelin proteins at sites of active remyelination [111]. 

Interestingly, TDP-43 aggregates have been detected in spinal cord OLs from ALS patients and strongly correlated with MN loss [103]. Accordingly, the presence of TDP-43 aggregates in OLs has been recently associated with impaired MBP mRNA translation and myelin abnormalities in ALS patients, particularly those carrying C9orf72 mutations, not merely representing a consequence of MN loss but instead directly promoting axonal damage [91]. Notably, a very elegant study reported progressive motor deficits in mature OL-specific TDP-43 knockout mice, caused by loss of mature OLs and defective myelination [112]. Very relevant, transcriptomic studies have been performed on the spinal cords of mutant mice carrying a patient-specific point mutation of TDP-43, displaying typical features of ALS including motor and cognitive symptoms [113]. Intriguingly, results have correlated improved behavioral function with increased mRNA levels of myelin-related genes, suggesting that favoring remyelination may indeed represent a promising strategy to reduce the risk of developing neurological deficits [113].

Myelin damage has also been reported in association with cytoplasmic inclusions of mutant FUS aggregates in mature OLs [102]. Consistently, mice expressing a truncated form of FUS, characterized by toxic gain-of-function and cytoplasmic mislocalization of this protein occurring in both MNs and OLs, showed motor deficits and progressive MN degeneration [114]. Transcriptomic analysis of the spinal cord of these mutant FUS-bearing mice unveiled downregulation of myelin-related genes as compared to controls, accompanied by the alteration of OL number in ventral horn WM and myelination defects [114]. Interestingly, MN-specific CRE-mediated reversal of FUS mutation significantly reduced MN loss, although it was not able to prevent myelin defects and motor symptoms [114]. These data suggest that, in the presence of FUS mutations, OL dysfunction develops independently of MN damage and primarily contributes to functional deficits [114]. Accordingly, a very recent work described the crucial role of FUS in controlling myelination by exploiting conditional OL-specific FUS knockout mice [115]. Genetic ablation of FUS from OLs resulted in hypermyelination without significant alteration of the total number of OPCs and mature OLs. The increased myelin thickness was found to be a consequence of enhanced synthesis of cholesterol and activation of Akt signaling [115].

#### 3.3.3. Altered Expression of OL Differentiation Regulators

Several intrinsic and extrinsic factors can increase OL vulnerability and impair OPC maturation, independently of the presence of disease-related mutant genes. In particular, OL differentiation is finely regulated by an intricate network of signaling cascades and intracellular molecular pathways [11,116]. Notably, all these factors could be dysregulated in ALS, leading to OL dysfunction and remyelination failure. Despite current research on altered OL differentiation in ALS still being at the beginning, unraveling the dysregulated factors contributing to maturation failure may help in defining novel therapeutic targets.

In this respect, Id2 and Notch are two important factors working as inhibitors of OL differentiation [117,118], which also received attention in the context of MS [119,120,121]. Recent data showed that OPC-specific conditional deletion of Id2 and Notch1 was not able to restore proper levels of MBP and MCT1 in the spinal cord of SOD1 G93A mice, and did not result in significant improvement of survival and motor function [122]. Despite Notch signaling being found to be dysregulated in the spinal cord of SOD1 G93A mice, no changes were observed in the levels of Notch ligands within OLs, suggesting that this pathway might not contribute to OL dysfunction [123]. However, it remains possible that targeting regulators of OL maturation and myelination other than Id2 and Notch1 could provide better results.

Another important regulator of OPC differentiation is the P2Y-like GPR17 receptor, which drives the initial steps of this process, and it is then downregulated to allow the final maturation of OLs (Section 2.1; [124]). Interestingly, GPR17 protein levels have been found to be pathologically increased in the lumbar spinal cord of SOD1 G93A mice at both pre-symptomatic and late symptomatic stages of the disease as compared to littermates expressing the non-mutant form of SOD1 [96]. Higher GPR17 levels at early pre-symptomatic stages are coherent with enhanced OPC reactivity aimed at replacing dysfunctional OLs, while prolonged upregulation at terminal stages has been associated with OPC differentiation blockade [96]. Previous studies in other experimental models of neurodegeneration and oligodendroglia pathology suggest that targeting GPR17 may be a good strategy to support OPC function (as recently reviewed in [124]). Accordingly, in vitro results suggest that GPR17 may represent a promising pharmacological target to restore proper oligodendroglial differentiation capability also in SOD1 G93A mice [96]. However, an in vivo validation of this approach in mouse models of ALS is still missing.

#### 3.3.4. Neuroinflammation

The massive activation of microglia and astrocytes plays a central role in neuroinflammation during the pathogenesis of ALS [84,85]. The presence of ALS-related mutant genes and protein aggregates within these cells was demonstrated to induce a detrimental phenotype, directly triggering the death of MNs [100,125,126,127,128]. For example, microglia-specific OPTN deficiency has been reported to induce the pro-inflammatory activation of these cells through a RIPK1-dependent mechanism distinct from necroptosis, suggesting that OPTN deficiency in microglia and OLs may contribute to axonal degeneration by different cell type-specific mechanisms [109]. The MN-toxic phenotype of microglia was found to be restricted to the symptomatic phase of the disease, whereas, at early pre-symptomatic stages, these immune cells were shown to acquire a protective activation state in an attempt to counteract early pathological events [129]. In this respect, a recent study exploited spatial transcriptomic approaches to demonstrate that microglial phenotypic switch in the spinal cord of SOD1 G93A mice and ALS patients occurs already at pre-symptomatic disease stages in proximity of MNs, thus preceding and probably inducing the harmful activation of astrocytes. In these conditions, the loss of the beneficial properties of microglia was found to be triggered by alterations of TREM2-dependent phagocytic pathways [130].

Notably, the mutual interplay between OLs and CNS-resident neuroinflammatory cells is essential for myelin maintenance and repair [69,131,132,133]. During developmental myelination, microglia are necessary to correctly induce OPC differentiation and therefore axon ensheathing, whereas, during adulthood, these immune cells were found to tightly regulate the number of quiescent OPCs [134]. Interestingly, microglial cells were shown to control myelinogenesis through the phagocytosis of excessive and ectopic myelinated tracts, with a mechanism resembling the well-known synaptic pruning crucial for neuronal development [135]. Like microglia, astrocytes extensively interact with OLs to regulate myelin homeostasis during development and adulthood, for example by providing lipids and other nutrients necessary for myelination [61,136], and by controlling myelin thickness and paranodal length to optimize axonal conduction speed [137].

In response to myelin injury, microglia were shown to acutely respond by acquiring a pro-resolving phenotype to limit disease progression and to promote lesion repair by phagocytosing myelin debris [138] and by releasing regenerative factors, directly [139] or via extracellular vesicles [140]. Conversely, at chronic disease stages both in experimental models of focal demyelination and cerebral ischemia, microglial cells become prominently pro-inflammatory and lose their beneficial functions, contributing to disease exacerbation and contrasting myelin regeneration [141,142]. A detrimental phenotype of astrocytes is also induced by pro-inflammatory microglia, further counteracting any regenerative attempt and promoting damage to both neurons and OLs [140,143]. 

On this basis, the pro-inflammatory activation of microglia and astrocytes, which characterizes the late symptomatic stages of ALS [144,145,146,147], may contribute to create a hostile local environment, hindering myelin regeneration and promoting OL and MN degeneration. Moreover, altered microglial activity at early pre-symptomatic phases [148] may negatively impact on OLs, leading to their dysfunction.

#### 3.3.5. Dysregulated Energy and Lipid Metabolism

An important hallmark of ALS is the presence of energy metabolism alterations, which were shown to correlate with disease progression and contribute to neurodegeneration [149]. Indeed, a proper energy homeostasis is essential to sustain the primary function of MNs, namely axonal transmission, requiring a constant ATP supply [150]. However, MNs are not capable of self-sustaining their metabolic necessities, and the contribution of OLs, which provide substrates for neuronal ATP synthesis, is fundamental [151]. Consequently, any interruption of this continuous energy provision inevitably compromises neuronal integrity [60].

OLs themselves are characterized by an extremely high energy demand to both efficiently produce myelin sheaths and completely fulfill axonal metabolic needs [152]. Thus, OLs are even more vulnerable than MNs to any condition affecting energy homeostasis, which quickly triggers their degeneration and thus contributes to demyelination. In addition, an efficient remyelination of CNS lesions by OPCs would never be possible without an adequate energetic support [152]. 

Interestingly, many alterations in OL metabolism have been reported in demyelinating conditions. These include mitochondrial dysfunction, which leads to reduced production of ATP and increased levels of reactive oxygen species (ROS), both contributing to oligodendroglial energy failure [153]. In primary demyelinating conditions like MS, dysfunctional mitochondria were detected in proximity of demyelinated areas, which have been proposed to represent early events preceding and promoting OL death [152]. Accordingly, axonal demyelination and motor disability, mimicking the pathological characteristics of progressive forms of MS, have been reported in transgenic mice carrying mitochondrial DNA double strand breaks specifically in myelinating OLs, linking mitochondrial dysfunction and myelin abnormalities [154]. Of note, mitochondrial dysfunction was shown to be widely diffused in both familial and sporadic forms of ALS [149]. Hence, enhancing mitochondrial function in OLs may help to counteract disease progression in demyelinating diseases as well as in ALS.

Another crucial aspect of OL metabolism is the efficient production of lipids, mainly cholesterol and sphingolipids, necessary to constitute myelin sheaths [155,156]. Impaired cholesterol biosynthesis was demonstrated to dampen myelin production during CNS myelinogenesis and remyelination [157,158]. Notably, alterations in myelin lipid composition have also been reported in SOD1 G93A rats, suggesting that lipid production in OLs may also be impaired in ALS [94]. On this basis, restoring lipid homeostasis in OLs represents an interesting therapeutic opportunity to foster myelin repair, also considering that this is a common downstream mechanism of many remyelinating drugs [155,159]. Accordingly, genes involved in cholesterol biosynthetic pathways were found among the top upregulated ones in the corpus callosum of cuprizone-fed mice during the spontaneous remyelination phase [160]. Moreover, dietary cholesterol supplementation significantly promoted OPC maturation and remyelination in different experimental demyelination models [161]. Interestingly, even the pro-myelinating role of microglia was recently shown to rely, at least in part, on the transfer of cholesterol precursors to OLs [158]. Although to our knowledge no studies have investigated so far lipid alterations in OLs in an ALS-related context, addressing how these pathological alterations may contribute to myelin degeneration in ALS could be very relevant for future therapeutic approaches.

#### 3.3.6. Oxidative Stress

Many of the pathological processes described in the previous paragraphs, including the harmful activation of microglia and astrocytes and the impairment of mitochondrial metabolism, contribute to increase the oxidative damage in OLs [162]. The resulting condition of extreme oxidative stress, which represents one of the main characteristics observed in both ALS patients and animal models (recently reviewed in [163]), has been demonstrated to negatively affect OL integrity, at least in two different ways. First, this oxidative charge is particularly detrimental for proliferating early OPCs, due to their reduced expression of protective antioxidant enzymes as compared to mature OLs, leading to the loss of the reserve pool of progenitors required to initiate myelin repair [164]. Second, the terminal maturation of the surviving OLs and the consequent remyelination were demonstrated to be impaired by prolonged exposure to ROS and other reactive radicals [165], which may concur to the OL differentiation impairment described at chronic disease stages.

Given the role of oxidative stress in counteracting efficient myelin regeneration, the use of radical scavengers represents a good therapeutic strategy, targeting a common downstream effector pathway of different pathogenetic mechanisms [163]. Accordingly, one antioxidant agent, namely edaravone, has already been approved for ALS treatment, and many other molecules are currently in development (refer Section 4). However, it should also be considered that, to date, antioxidant therapy has been proven poorly effective, possibly because of the late time of compound administration, which mainly occurs at already symptomatic stages when oxidative damage becomes very difficult to be completely reverted. On this basis, approaches aimed at correcting the upstream processes involved in the overproduction of reactive species, i.e., by promoting a pro-resolving microglial phenotype or by fostering mitochondrial metabolism, might result in a more efficient protection of OLs [166,167].

**Figure 3:**
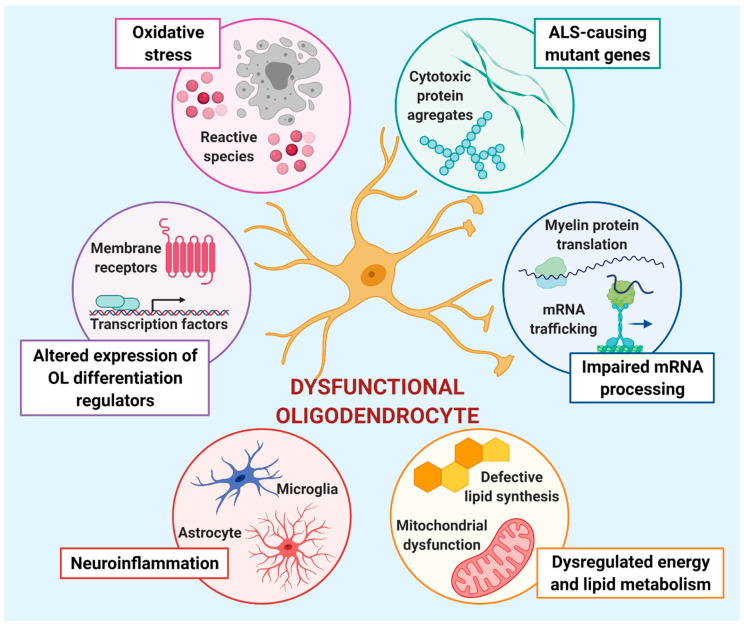
Mechanisms contributing to oligodendrocyte (OL) dysfunction and degeneration in ALS. The presence of ALS-related mutant genes in OLs has been shown to produce detrimental effects by inducing alterations of their normal function or by the formation of cytotoxic protein inclusions. Moreover, given that several ALS-linked genes are involved in mRNA processing, impaired trafficking of myelin-associated mRNAs from the nucleus to cell processes and their translation have been implicated in remyelination failure. Besides genetic mutations, other factors dysregulated in ALS may contribute to the generation of dysfunctional OLs. These include the altered expression of key regulators of OL differentiation, i.e., transcription factors and membrane receptors, and abnormalities in OL metabolism, impacting the mitochondrial energy production and the synthesis of lipids required for myelin repair. Proper OL differentiation may be also affected by the harmful inflammatory environment, characterized by the overactivation of microglia and astrocytes. All these detrimental processes also concur to put OLs in a condition of extreme oxidative stress, further impairing their functions.

Taken together, the results described in the previous paragraphs suggest the existence of different mechanisms potentially contributing to OL pathology and dysfunction (Figure 3), which may also represent important therapeutic targets in ALS.

## 4. Emerging Therapeutics Targeting OL Dysfunction of Potential Utility for Myelin Regeneration in ALS

An increasing body of evidence suggests that OL dysfunction precedes the onset of ALS symptoms and concurs to aggravating MN degeneration. Such defects are due to the impaired capacity of OPCs to differentiate into fully functional mature OLs, with consequent production of dystrophic immature cells unable to myelinate and to provide metabolic support to MNs (Section 3). On these bases, strategies aimed at implementing the metabolic and remyelinating abilities of OPCs may help to preserve MNs from degeneration, opening new therapeutic perspectives for the treatment of ALS.

To date, only few studies have been conducted to analyze the effects of remyelinating molecules in an ALS-specific context. Nonetheless, many drugs tested in ALS for their effects on neuroinflammation or other pathological processes could also directly influence OPC maturation and myelin production, thus being useful for this additional application. Furthermore, given the numerous similarities in the pathogenesis of ALS with other neurological disorders, including progressive forms of MS, molecules currently under evaluation for the treatment of different neurological diseases may also provide relevant benefits in ALS patients [2,3]. In the paragraphs below, we focused our attention on molecules of potential interest for ALS treatment for their remyelinating effect or capability to enhance OL energy metabolism. Of note, for some of these compounds, there are already available data supporting their protective role in experimental models of ALS or patients.

### 4.1. Clemastine

Clemastine is a first-generation anti-histaminergic drug, currently employed in clinical practice to relieve allergic symptomatology, whose administration may represent a very interesting treatment to address OL dysfunction in ALS. Preclinical data indeed showed beneficial effects of chronic clemastine treatment in SOD1 G93A mice, which are mainly related to the ability of this drug to suppress inflammatory activation of microglia through the inhibition of histaminergic H1 receptors [168]. Of note, clemastine was able to delay symptoms onset and to prolong overall survival of SOD1 G93A mice only when administrated at a pre-symptomatic disease stage [169], a phase during which OL dysfunction critically develops ([101]; Section 3). Interestingly, besides its anti-histaminergic actions on microglia, clemastine has a potent pro-differentiating effect on OPCs, which is mainly mediated by the blockade of muscarinic M1 receptor [170]. Notably, clemastine administration has been shown to efficiently support remyelination in vivo in various experimental models characterized by demyelination, leading to behavioral improvement [171,172,173,174]. This latter finding is of particular relevance because its efficacy as a remyelinating agent has also been confirmed in clinical studies on MS patients [175]. Thus, evaluating if clemastine treatment is able to restore proper OPC maturation in SOD1 G93A mice and other ALS experimental models might set the basis for testing this drug on ALS patients.

### 4.2. Bexarotene

Another interesting molecule is bexarotene, a retinoic X receptor-gamma (RXR-γ) agonist exerting beneficial effects in SOD1 G93A mice, including enhanced motor performance, prolonged overall survival and reduced loss of MNs [176]. Notably, activation of RXR-γ signaling has been previously demonstrated to enhance remyelination by directly promoting OL differentiation [177]. This effect was shown to require RXR-γ heterodimerization with a nuclear vitamin D receptor [178]. Bexarotene was also able to enhance the myelin debris clearance capacity of human monocytes isolated from MS patients, which is essential to promote efficient remyelination [179]. Of note, a first clinical trial has been performed in MS patients and recently concluded (EudraCT 2014-003145-99), whose results are not available yet but may help to better define the feasibility of bexarotene treatment also in human specimens. However, a detailed evaluation of bexarotene effects on OL dysfunction in ALS models has never been performed and will be crucial for the potential application of this drug in ALS patients. 

### 4.3. Cannabinoids

The endocannabinoid system also represents a putative pharmacological target to sustain OL functions in ALS. Indeed, cannabinoid CB1 and CB2 receptors have been positively implicated in different phases of OPC-mediated remyelinating response, including proliferation, migration, and differentiation (reviewed in [180]). Accordingly, blocking the enzymatic degradation of 2-arachidonylglycerol (2-AG), the main endogenous ligand of cannabinoid receptors, promoted OL differentiation and remyelination in a model of progressive demyelination [181]. Administration of Δ^9^-Tetrahydrocannabinol (THC), one of the main bioactive components contained in the plant *Cannabis sativa*, enhanced postnatal oligodendrogenesis and myelination by activating both CB1 and CB2 receptors [182]. More relevant for CNS therapies, treatment with the non-selective CB1/CB2 agonist WIN55,212-2 has been demonstrated to effectively enhance myelin repair after experimental stroke [183]. Interestingly, alterations of the cannabinoid system have been detected in preclinical models and ALS patients [184,185,186], and cannabinoid-based therapies are already diffused in ALS clinical practice to manage pain and spasticity associated with disease progression [187,188]. Furthermore, activation of cannabinoid receptors was demonstrated to exert neuroprotective and immunomodulatory effects in ALS models. In SOD1 G93A mice, treatment with WIN55,212-2, starting from disease onset, ameliorated muscular strength and MN viability, extending the overall survival [189]. In this seminal paper, it was proposed that the protective effects observed were independent from CB1 activation, as genetic knockout of this receptor in SOD1 G93A mice significantly extended mice lifespan [189]. These results shifted the focus on CB2 receptor as a more promising target in ALS. In this respect, another group reported that WIN55,212-2, and, more markedly, the selective CB2 agonist HU-308, were able to effectively improve rotarod performance, preserve MNs and attenuate neuroinflammation markers in the spinal cord of TDP-43 A315T transgenic mice [190]. However, it remains to be evaluated if these compounds exert beneficial effects also on oligodendroglial cells in an ALS-specific context.

### 4.4. Edaravone

A potential remyelinating activity has also been recently proposed for edaravone, an antioxidant molecule representing one of the only two drugs currently approved by the Food and Drug Administration (FDA) for ALS treatment [191]. Indeed, it is known that oxidative stress dampens OPC-mediated response to injury, and blocking this process with edaravone resulted in effective remyelination [192,193]. Interestingly, edaravone administration was found to exert protective effects in a murine model of Alzheimer’s disease with chronic cerebral hypoperfusion, by preserving WM integrity and directly promoting OPC proliferation and maturation [194]. Notably, a phenotypic screening performed on a library of 2000 molecules identified edaravone among the most promising candidates for myelin repair strategies, based on its positive effects on OL metabolism, differentiation and myelination capability in a battery of in vitro assays [195]. In the same study, the functional groups responsible for the remyelinating activity of edaravone were also defined [195], paving the way for the development of optimized derivative compounds able to efficiently counteract OL dysfunction in demyelinating diseases and in ALS.

### 4.5. GPR17 Receptor Modulators

The P2Y-like GPR17 receptor represents a promising candidate target for novel remyelinating therapies. This was proposed based on its prominent role in fine-tuning the OPC differentiation program and on its aberrant overexpression in different neuropathological conditions, which has been related to impaired maturation and reduced remyelination capabilities of OPCs surrounding CNS lesions (refer Section 2; reviewed in [124]). However, a still unsolved question is whether a GPR17 agonist or antagonist would be a more promising therapeutic strategy [124]. On one end, blockade of GPR17 by antagonists may help to counteract aberrant upregulation of the receptor in immature OLs, allowing them to complete their maturation. Accordingly, exposure to the non-selective GPR17 antagonist HAMI3379 was able to stimulate OL maturation in vitro [196]. Moreover, the non-selective antagonist pranlukast enhanced OPC differentiation and remyelination after lysolecithin-induced focal demyelination [197]. Similarly, it was recently shown that the use of another GPR17 antagonist, montelukast, promotes remyelination in experimental models of stroke [198] and optic nerve injury [199]. Very relevant for ALS, exposure to montelukast significantly restored the differentiation defects of primary cultured OPCs isolated from SOD1 G93A mice, emerging as a good candidate to counteract OL dysfunction in this disease [96]. Further studies are currently ongoing to evaluate the efficacy of this approach also in vivo in experimental ALS models, which will provide important data also for the clinical feasibility of this strategy. In this respect, montelukast is particularly interesting, being an already FDA-approved and marketed drug quickly translatable into the clinics. 

Another possible strategy to correct OL dysfunction by targeting GPR17 could be represented by the use of receptor agonists, which may promote GPR17 expression in early OPCs, pushing them to start differentiation. Accordingly, treatment with the endogenous GPR17 agonist UDP-glucose significantly promoted OPC maturation [32] and has been shown to trigger receptor desensitization and internalization at the peak of its expression [200,201], a crucial step to allow terminal maturation of OLs [36]. In both cases, the use of GPR17 agonist resulted in a significant amelioration of OL differentiation and myelination capabilities. Indeed, the selective GPR17 agonist Asinex 1 promoted OPC maturation and myelination of dorsal root ganglion (DRG) neurons in vitro [202]. Of note, a recent in vivo study showed that the treatment with another selective agonist, named galinex, resulted in protection against EAE symptoms exacerbation [203]. 

To reconcile these apparently opposing results, it could be postulated that different ligands may exert their beneficial effect by influencing distinct moments of OL development. Agonist compounds are supposed to foster early OPCs to undertake differentiation in the initial phases of the remyelinating response. On the other end, antagonists may act at later stages on GPR17-overexpressing immature OLs, pushing them to terminal maturation by ablating the inhibitory action of the receptor. Accordingly, both GPR17 agonists and antagonists showed promising results in many preclinical studies in vitro and in vivo [124]. Given all these good premises, the further development and optimization of novel selective compounds, together with well-designed in vivo studies will help to set the basis for testing GPR17 ligands in clinical trials.

### 4.6. Selective Estrogen Receptor Modulators

Selective estrogen receptor modulators, including tamoxifen and its derivatives, also provide an interesting possibility for ALS treatment. Indeed, a recent population-based case-control study associated tamoxifen treatment with reduced ALS risk in humans [204], and some beneficial effects of this drug have been already reported in a phase II clinical trial on a small cohort of ALS patients without SOD1 and FUS mutations [205]. These protective effects have been related with the impact of tamoxifen on the autophagic process, resulting in enhanced clearance of TDP-43 protein aggregates [206]. Nonetheless, a potent remyelinating activity of tamoxifen has been described in a toxin-induced demyelination model [207]. This pro-remyelinating property has been confirmed also for another compound belonging to the same class, namely bazedoxifene. Interestingly, the protective effect of this molecule was demonstrated to be independent from estrogen receptors. Indeed, it was shown to exert its protective function through the involvement of key enzymes regulating cholesterol biosynthesis [208], an essential process for myelin production that is now stealing the attention for the development of novel remyelinating strategies [160,163,165,166]. Notably, also other remyelinating molecules, including tamoxifen and the aforementioned clemastine, were found to partially act by modulating various steps of the cholesterol biosynthetic pathway [159], further corroborating the importance of strategies targeting this biochemical process to foster myelin repair. Based on these premises, a phase II clinical trial is actually in the recruitment phase and will evaluate the efficacy of bazedoxifene as a remyelinating agent in MS patients (ClinicalTrials.gov, accessed on 5 February 2021, Identifier: NCT04002934), hopefully providing important data also for the treatment of OL dysfunction associated with ALS.

### 4.7. MD1003

As already mentioned, stimulating the biosynthesis of the lipidic components of myelin emerged as a promising strategy to improve remyelination. In this respect, an interesting approach could be represented by the therapeutic treatment with biotin, an important co-enzyme required for the proper activity of several carboxylases involved in cellular energetic metabolism [209]. Biotin-dependent enzymes include propionyl-CoA carboxylase, 3-methylcrotonyl-CoA carboxylase, and pyruvate carboxylase, providing intermediate metabolites for the tricarboxylic acid (TCA) cycle, and acetyl-CoA carboxylases, which regulate the production of malonyl-CoA utilized during fatty acids synthesis [209]. Based on these functions, biotin might have a potential impact on remyelination by sustaining the production of ATP and lipids in OLs. Accordingly, biotin was recently demonstrated to protect primary rat OL cultures from metabolic stress induced by glucose deprivation, to improve their capacity of ensheathing artificial nanofibers and to enhance their ATP production [210]. Treatment with high-dose pharmaceutical-grade biotin, named MD1003, resulted in significant functional improvement in a proof-of-concept pilot study performed on progressive MS patients [211]. These promising data were partially confirmed by two subsequent clinical studies showing that the treatment was generally well tolerated and capable of counteracting MS-related disability in a subset of patients [212,213]. Notably, a pilot study has been performed to evaluate the potential impact of MD1003 in ALS patients, whose results indicate that, also in this different pathological context, the treatment was safe and well tolerated. However, no certain conclusions could be drawn in terms of efficacy, due to the small number of patients enrolled and to the imbalance between the experimental groups [214]. Further studies on a larger cohort of ALS patients are thus certainly needed to assess the potential application of MD1003 for ALS therapy. Despite the great expectations produced by such favorable clinical data on safety and efficacy, recent negative results from a phase III trial, conducted to assess the efficacy of MD1003 on a disability outcome on a large cohort of non-relapsing progressive MS patients, may dampen the enthusiasm [215]. In fact, the previously shown beneficial effects of MD1003 were not replicated by this study. An additional issue is represented by the potential interference of this drug with diagnostic laboratory tests employing biotinylated antibodies, which may lead to inaccurate results representing an important safety risk for patients [215]. 

### 4.8. RNS60

An emerging therapeutic approach in neurological diseases characterized by neuroinflammation and impaired energetic metabolism, including ALS, is represented by RNS60, a physically modified solution obtained by exposing normal saline to Taylor-Couette-Poiseuille (TCP) flow under elevated oxygen pressure. This peculiar procedure allows for generating oxygen-filled charge-stabilized nanobubbles dispersed within an aqueous vehicle, which are accounted for the beneficial effects of this novel drug [216]. The proposed mechanism for the biological activity of RNS60 involves the direct activation of the phosphoinositide 3-kinase (PI3K)-Akt pathway in microglial cells, which resulted in the suppression of nuclear factor-kappa B (NF-kB) and consequent inhibition of pro-inflammatory activity [216]. By counteracting detrimental excessive activation of microglia in vivo, RNS60 was shown to exert significant neuroprotective effects and to improve cognitive and motor function in experimental models of PD [217], Alzheimer’s disease [218], and traumatic brain injury [219]. In parallel to the anti-inflammatory properties of RNS60, an additional mechanism of action involving enhanced ATP production in target cells has been demonstrated, which is related to the ability of this drug to directly fuel the mitochondrial electron transport chain and to promote mitochondrial biogenesis dependent on PI3K-cAMP responsive element binding protein (CREB)-mediated upregulation of peroxisome proliferator-activated receptor gamma coactivator 1-alpha (PGC1α) [220,221,222]. 

RNS60 has been demonstrated to exert significant beneficial effects on primary OL cultures, protecting cells from metabolic stress induced by glucose-nutrient deprivation, enhancing their bio-energetic activity in both basal and stressed conditions, and promoting OPC differentiation into mature MBP-expressing cells [223]. RNS60 stimulated the expression of myelin genes (*Mbp*, *Mog*, and *Plp*) in primary OLs and mixed glial cultures by PI3K-dependent upregulation of CREB, a transcription factor regulating the activity of myelin-specific gene promoters [224]. On these bases, RNS60 seems to retain the capacity to positively affect remyelination by simultaneously activating energy metabolism in OL lineage cells and reducing inflammatory properties of microglia, which are known to negatively impact myelin repair [133]. Interestingly, RNS60 showed protective effects also in vivo in the EAE model of MS, by acting on both peripheral and CNS-resident immune cells and by preserving myelin structures, significantly preventing disease exacerbation [225,226]. A significant therapeutic efficacy of RNS60 has also been observed in mutant SOD1 mice. In this murine model of ALS, this treatment was able to delay the onset of muscle weakness and paralysis and to prolong survival interval. Authors demonstrated that the positive effects observed were due to its anti-inflammatory action on microglia, astrocytes, and circulating immune cells and to the protective role exerted on MNs, myelinating Schwann cells, and muscles [227]. These promising preclinical data prompted the interest for a rapid translation of RNS60 into the clinical management of ALS. A first pilot clinical trial, already concluded, confirmed the feasibility and tolerability of this treatment in ALS patients [228], paving the way to subsequent larger studies. Indeed, two different phase II clinical trials are currently ongoing to evaluate the efficacy of RNS60 in ALS. The first one (ClinicalTrials.gov, accessed on 5 February 2021, Identifier: NCT03456882) primarily aims at analyzing the impact of RNS60, administered in association with riluzole, on peripheral blood pharmacodynamic biomarkers (proteins or RNAs) that could be helpful to monitor patient’s response to therapy; the effects of the treatment on functional disability, survival, and quality of life will also be assessed. Another active study (ClinicalTrials.gov, accessed on 5 February 2021, Identifier: NCT02988297) is focused on the impact of nebulized RNS60, a novel formulation which is poorly invasive for patients, on ALS-related disability and mortality.

### 4.9. CNM-Au8

Recently, the biocatalytic activity of gold nanoparticles has been demonstrated [229], which could be very relevant to enhance the activity of ALS dysfunctional OLs, characterized by elevated oxidative stress and impaired energy metabolism. Indeed, gold nanoparticles were shown to enhance the oxidation of NADH to NAD^+^ [230], a key step for providing the energetic metabolites required for myelin synthesis [59], which was found defective in the spinal cord of ALS patients [231]. Other important catalytic properties have been shown for gold nanoparticles, including peroxidase, oxidase, catalase, and superoxide dismutase-like activities [232,233], which could also provide potential benefits for ALS patients. Among the different formulations available, the aqueous suspension of clean-surfaced, faceted gold nanocrystals, termed CNM-Au8, is characterized by high catalytic activity and favorable safety profile [234]. The impact of CNM-Au8 on OLs and remyelination has been evaluated using different experimental systems. In oligodendroglial cell cultures, exposure to CNM-Au8 was able to improve cell differentiation and to elevate the glycolytic activity, resulting in increased extracellular levels of lactate [234]. These results are particularly relevant for ALS, in which an impairment of lactate release by OLs has been clearly linked to degeneration of MNs (refer Section 3). Notably, therapeutic treatment with CNM-Au8 was also able to significantly enhance OL differentiation and remyelination after cuprizone-induced demyelination in vivo, leading to improved performance assessed by open field test and fine motor kinematic analyses [234]. Importantly, toxicologic studies and a phase I clinical trial (ClinicalTrials.gov, accessed on 5 February 2021, Identifier: NCT02755870) on healthy male and female volunteers have been already performed for CNM-Au8, demonstrating BBB permeability and favorable safety profile of this compound.

Based on these promising features, a phase II clinical trial (ClinicalTrials.gov, accessed on 5 February 2021, Identifier: NCT03536559) is currently ongoing to evaluate the efficacy of CNM-Au8 as a remyelinating agent in relapsing-remitting MS patients with visual deficits. Results from this study are expected in 2021 and will clarify if a potential application of CNM-Au8 in patients affected by demyelination, including those with ALS, is also achievable.

To conclude, the following table recapitulates the most relevant findings regarding the impact of the aforementioned drugs on myelin repair and their relevance for a potential application to counteract OL dysfunction in ALS (Table 1).

## 5. Conclusions

Despite the increased understanding of the genetic, epigenetic, and environmental factors contributing to ALS [79], no effective treatment is currently available for this progressive and fatal neurodegenerative disorder. 

Recent findings have revealed an unexpected early role of OLs in the degeneration and death of MNs. Of note, OL damage and impaired maturation of OPCs in ALS share similarities to progressive forms of MS, where OL pathology and myelin deterioration eventually lead to neuronal death [235]. This has promoted the investigation of the potential mechanisms involved in OL alterations and various studies have identified diverse processes that could be, or have already been, the target of therapeutic interventions in ALS. As described in this review, ALS-causing genes have been shown to trigger OL degeneration, by generating mutant proteins which form toxic aggregates or causing a potential dysfunction of myelin-related mRNA processing. In addition, abnormalities of several key pathways regulating OL differentiation could also contribute to OPC maturation failure in ALS. Furthermore, a dysregulated energy metabolism in ALS was shown to correlate with disease susceptibility and progression [149]. In particular, the reduced metabolic and trophic support, specifically provided by OLs to neurons, was shown to critically compromise axonal integrity [60]. 

On this basis, novel strategies aimed at implementing the metabolic and remyelinating abilities of OPCs may help to preserve MNs from degeneration, opening new therapeutic perspectives for ALS treatment. Importantly, the complex pathophysiology of ALS suggests that novel drugs acting synergistically or combination therapies targeting different pathological processes simultaneously would be the “perfect” therapeutic approach [236,237]. Here, we have highlighted that some compounds already evaluated in preclinical models of ALS for their capability to counteract neuroinflammation or other pathological processes are also known to positively affect OPC maturation or OL energy metabolism. However, further studies are needed to prove their efficacy in a context close to human paradigms and to unravel the importance of OLs as a treatment target in ALS. In this respect, the generation of induced pluripotent stem cell (iPSC)-derived OLs from ALS patients emerges as a valuable model to better understand human disease mechanisms and to test drugs for evaluating their remyelination and metabolic potential [238]. 

Improvements in clinical trials are also considered fundamental to speed the translation of potential novel therapies into clinic [237]. This is also valid for regenerative/remyelinating agents, for which an appropriate trial design should take into account a correct stratification of patients, the right window of intervention (e.g., remyelinating drugs delivered too late may be ineffective) and the development of adequate outcome measures, in order to better determine target engagement and the effect on the disease. 

From this scenario, advances in the understanding of the pathogenetic mechanisms underpinning OL dysfunction during ALS disease progression, together with the optimization of pre-clinical and clinical trial design, will pave the way for a successful development of novel therapeutic agents for ALS. 

## Figures and Tables

**Table 1:** Promising candidate drugs to counteract OL dysfunction in ALS.

Drug	Mechanism of Action	Impact on Remyelination	Relevance for ALS
Clemastine	H1/M1 receptor antagonist	Promotes OPC differentiation [170,171,172,173,174,175]	Pre-symptomatic treatment delays symptoms onset and prolongs survival in SOD1 G93A mice [168,169]
Bexarotene	RXR-γ agonist	Promotes OPC differentiation and clearance of myelin debris by macrophages [177,178,179]	Improves motor performance, prolongs survival and reduced loss of MNs in SOD1 G93A mice [176]
WIN55,212-2	CB1/CB2 receptors agonist	Promotes OPC differentiation and remyelination after experimental stroke [183]	Improves motor function, increases MN viability, and extends survival in mutant SOD1 and TDP-43 mice [189,190]
HU-308	CB2 agonist	Unknown	Improves motor function and reduces neuroinflammation in TDP-43 A315T mice [190]
Edaravone	Radical scavenger	Protects OPCs from oxidative stress, favoring their survival, proliferation and maturation [193,194,195]	FDA-approved drug for ALS treatment [191]
Montelukast	GPR17 receptor antagonist	Promotes OPC differentiation and remyelination in vivo [198,199]	Rescues differentiation defects of OPCs isolated from SOD1 G93A mice spinal cord [96]
Asinex1 and Galinex	GPR17 agonists	Promote OPC differentiation and myelination in vitro and delay EAE onset [202,203]	Unknown
Tamoxifen	Selective Estrogen Receptor Modulator	Promotes OPC differentiation and remyelination [207]	Treatment correlates with reduced ALS risk in humans and attenuates disability progression in ALS patients [204,205]
Bazedoxifene	Selective Estrogen Receptor Modulator	Enhances myelin production by stimulating cholesterol biosynthesis in OLs [208]	Unknown
MD1003	Co-enzyme involved in energy metabolism	Fosters the production of ATP and fatty acids in OLs [209,210]	Safe and well-tolerated in a pilot trial on ALS patients [214]
RNS60	Stimulates mitochondrial biogenesis and oxidative phosphorylation	Stimulates ATP synthesis and protects OLs from metabolic stress [223,224]	Delays motor symptoms onset and prolongs survival of SOD1 G93A mice and results safe and well-tolerated in a pilot trial on ALS patients [227,228]
CNM-Au8	Biocatalytic activity	Improves OPC differentiation and elevates their glycolytic activity, resulting in increased extracellular levels of lactate [234]	Unknown

## Data Availability

No new data were created or analyzed in this study. Data sharing is not applicable to this article.
